# PRMT1 enhances oncogenic arginine methylation of NONO in colorectal cancer

**DOI:** 10.1038/s41388-020-01617-0

**Published:** 2021-01-08

**Authors:** Xin-Ke Yin, Yun-Long Wang, Fei Wang, Wei-Xing Feng, Shao-Mei Bai, Wan-Wen Zhao, Li-Li Feng, Ming-Biao Wei, Cao-Litao Qin, Fang Wang, Zhi-Li Chen, Hong-Jun Yi, Yan Huang, Pei-Yi Xie, Taewan Kim, Ying-Nai Wang, Jun-Wei Hou, Chia-Wei Li, Quentin Liu, Xin-Juan Fan, Mien-Chie Hung, Xiang-Bo Wan

**Affiliations:** 1 Guangdong Provincial Key Laboratory of Colorectal and Pelvic Floor Diseases, The Sixth Affiliated Hospital, Sun Yat-sen University, Guangzhou, Guangdong 510655 PR China; 2 Department of Radiation Oncology, The Sixth Affiliated Hospital, Sun Yat-sen University, Guangzhou, Guangdong 510655 PR China; 3 Department of Gastroenterology, The Seventh Affiliated Hospital, Sun Yat-sen University, Shenzhen, Guangdong 518107 PR China; 4 Department of Pathology, The Sixth Affiliated Hospital, Sun Yat-sen University, Guangzhou, Guangdong 510655 PR China; 5 Department of Radiology, The Sixth Affiliated Hospital, Sun Yat-sen University, Guangzhou, Guangdong 510655 PR China; 6 Base for International Science and Technology Cooperation, Carson Cancer Stem Cell Vaccines R&D Center, International Cancer Center, Shenzhen University Health Science Center, Shenzhen, 518055 PR China; 7 The Ohio State University Comprehensive Cancer Center, Columbus, OH 43210 USA; 8 Department of Molecular and Cellular Oncology, The University of Texas MD Anderson Cancer Center, Houston, TX 77030 USA; 9 Institute of Biomedical Sciences, Academia Sinica, Taipei, 11529 Taiwan; 10 Institute of Cancer Stem Cell, Dalian Medical University, Dalian, Liaoning 116044 PR China; 11 State Key Laboratory of Oncology in South China, Cancer Center, Sun Yat-sen University, Guangzhou, Guangdong 510060 PR China; 12 Graduate Institute of Biomedical Sciences and Research Centers for Cancer Biology and Molecular Medicine, China Medical University, Taichung, 404 Taiwan; 13 Department of Biotechnology, Asia University, Taichung, 413 Taiwan; 14 Department of Medical Engineering, The Sixth Affiliated Hospital, Sun Yat-sen University, Guangzhou, Guangdong 510655 PR China

**Keywords:** Colorectal cancer, Methylation, Prognostic markers

## Abstract

Arginine methylation is an important posttranslational modification catalyzed by protein arginine methyltransferases (PRMTs). However, the role of PRMTs in colorectal cancer (CRC) progression is not well understood. Here we report that non-POU domain-containing octamer-binding protein (NONO) is overexpressed in CRC tissue and is a potential marker for poor prognosis in CRC patients. NONO silencing resulted in decreased proliferation, migration, and invasion of CRC cells, whereas overexpression had the opposite effect. In a xenograft model, tumors derived from NONO-deficient CRC cells were smaller than those derived from wild-type (WT) cells, and PRMT1 inhibition blocked CRC xenograft progression. A mass spectrometry analysis indicated that NONO is a substrate of PRMT1. R251 of NONO was asymmetrically dimethylated by PRMT1 in vitro and in vivo. Compared to NONO WT cells, NONO R251K mutant-expressing CRC cells showed reduced proliferation, migration, and invasion, and *PRMT1* knockdown or pharmacological inhibition abrogated the malignant phenotype associated with NONO asymmetric dimethylation in both *KRAS* WT and mutant CRC cells. Compared to adjacent normal tissue, PRMT1 was highly expressed in the CRC zone in clinical specimens, which was correlated with poor overall survival in patients with locally advanced CRC. These results demonstrate that PRMT1-mediated methylation of NONO at R251 promotes CRC growth and metastasis, and suggest that PRMT1 inhibition may be an effective therapeutic strategy for CRC treatment regardless of *KRAS* mutation status.

## Introduction

Colorectal cancer (CRC) is the second most deadly cancer worldwide [1]. Neoadjuvant chemoradiotherapy and total mesorectal excision surgery have improved the outcome of locally advanced rectal cancer [2], and adding adjuvant chemotherapy decreases the rate of relapse in patients with locally advanced colon cancer [3]. However, the efficacy of the epidermal growth factor receptor (EGFR) inhibitor cetuximab is limited to patients with wild-type (WT) *KRAS* [4–6]. Moreover, distant metastasis is the leading cause of mortality in CRC; the 5-year survival of patients with metastasis is markedly lower than that of patients with nonmetastatic disease (90.0% vs 14.0%) [7]. Identifying novel therapeutic targets can provide a basis for the development of drugs that improve the outcome of metastatic CRC.

Non-POU domain-containing octamer-binding protein (NONO), also known as 54-kDa nuclear RNA- and DNA-binding protein (p54^nrb^), belongs to the *Drosophila* behavior/human splicing family and functions in a variety of physiologic processes including mRNA splicing [8], transcriptional regulation [9], DNA repair [10], and nuclear retention of defective RNA [11]. NONO also plays an important role in the regulation of malignant phenotypes in cancer cells [12]. In breast cancer, NONO increased nuclear sterol regulatory element-binding protein (SREBP)−1a protein stability and stimulated SREBP-1a–mediated transcription of lipogenic genes and lipid production, thus promoting breast cancer growth [13]. As a component of the cyclic (c)AMP signaling pathway, NONO was shown to interact with the long noncoding (lnc)RNAs LINC00473 and MetaLnc9 to facilitate transcription through cAMP-responsive element-binding protein (CREB) and CREB-regulated transcription coactivator to promote lung cancer growth and metastasis [14, 15]. However, the mechanism by which NONO regulates distant metastasis of cancer cells is not known.

NONO is posttranslationally modified in several ways including by phosphorylation, ubiquitination, and arginine methylation. As a cell cycle regulator, NONO is phosphorylated by cyclin-dependent kinase (CDK)1 at T412, T430, and T452 during mitosis for Pin1 binding [16]. In response to ultraviolet-induced DNA damage, NONO is ubiquitinated by RING finger protein (RNF)8 for degradation [17]. Polyubiquitination of NONO by F-box and WD repeat domain-containing (FBW)7a is triggered by glycogen synthase kinase (GSK)3β-mediated phosphorylation, which is involved in chromosomal rearrangement in cancer cells [18]. Moreover, coactivator-associated arginine methyltransferase (CARM)1-mediated NONO methylation regulates the nuclear retention of mRNAs containing inverted-repeat *Alu* elements under poly(I:C) treatment [19]. Additionally, NONO posttranscriptionally promotes the expression of S-phase-associated kinase (SKP)2 and E2F transcription factor (E2F)8 by directly binding to their mRNAs, leading to increased breast cancer cell proliferation [20].

Arginine methylation is a common posttranslational modification and it is catalyzed by protein arginine methyltransferases (PRMTs) that mainly occurs in nuclear proteins of eukaryotic cells. PRMTs—which are broadly classified as type I, II, or III—transfer methyl groups from S-adenosylmethionine (SAM) to the guanidine nitrogen of specific arginine residues of target proteins. Type I and II PRMTs catalyze the formation of ω-N^G^-monomethylarginine (MMA) as an intermediate; the former (including PRMT1, PRMT2, PRMT3, PRMT4, PRMT6, and PRMT8) stimulate the production of ω-N^G^, N^G^-asymmetric dimethylarginine (aDMA), while the latter (including PRMT5 and PRMT9) catalyze the formation of ω-N^G^, N^,G^-symmetric dimethylarginine (sDMA) [21]. In contrast, type III PRMT (PRMT7) only generates MMA [22]. Arginine methylation has been implicated in tumorigenesis and metastasis [23, 24]. For example, CARM1-mediated arginine methylation of BRG1-associated factor (BAF)155 was shown to control the expression of genes in the c-Myc pathway and regulate cell migration and metastasis in breast cancer [25]. We previously reported that PRMT1-mediated methylation of EGFR maintains its activation and promotes cell proliferation; increased EGFR methylation was correlated with worse survival in CRC patients who received cetuximab treatment [26]. Although PRMT1 has been reported to asymmetrically dimethylate splicing factor proline- and glutamine-rich (SFPQ)/PTB-associated splicing factor (PSF) to increase its association with mRNA without affecting complex formation with NONO in mammalian cells [27], it is not known whether NONO is a substrate of PRMT1.

In this study we demonstrate that NONO is overexpressed in CRC tissue and promotes CRC progression. We found that asymmetric dimethylation at R251 by PRMT1 was required for NONO-induced CRC cell proliferation, migration, and invasion. Inhibition of *PRMT1* by gene silencing or pharmacological treatment abrogated NONO methylation-associated increases in cell proliferation, migration, and invasion, regardless of *KRAS* mutation status. These results were confirmed in a CRC xenograft model treated with PRMT1 inhibitor. Thus, therapeutic strategies targeting PRMT1-mediated asymmetric arginine dimethylation of NONO are a promising treatment for CRC, irrespective of *KRAS* mutation status.

## Results

### NONO is overexpressed in CRC tissue and promotes cell proliferation, migration, and invasion

**Fig. 1:**
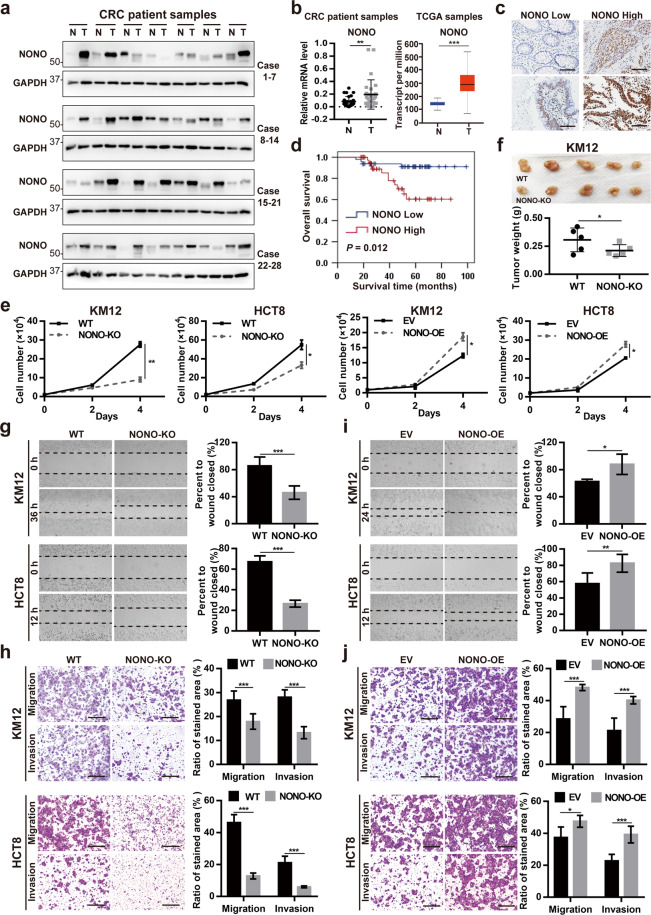
NONO is overexpressed in CRC tissue and promotes cell proliferation, migration, and invasion. **a** NONO protein is highly expressed in CRC tissue. NONO protein level was analyzed in 28 paired colorectal tumor (T) and adjacent normal (N) tissue samples by western blotting. Glyceraldehyde 3-phosphate dehydrogenase (GAPDH) was used as a loading control. **b** NONO mRNA level is higher in T than in N. NONO mRNA level was examined by qPCR in 29 paired tissue samples. U6 was used as an internal control (right panel). NONO mRNA expression level in TCGA N (*n* = 41) and T (*n* = 286) CRC samples (left panel). **c**, **d** Higher NONO protein level is correlated with shorter overall survival in CRC patients. The 93 patients were divided into NONO low (*n* = 48) and high (*n* = 45) subgroups (**c**) and subjected to Kaplan–Meier survival analysis (**d**). Scale bar, 1200 μm. **e** NONO overexpression promotes CRC cell growth. KM12 (1 × 10^4^) and HCT8 (2 × 10^4^) CRC cells were seeded on day 0 and counted on days 2 and 4. **f** NONO KO leads to a decrease in tumor size. KM12 WT or NONO KO cells (1 × 10^6^) were injected into nude mice (*n* = 5) and tumors were weighed on day 21. **g–j** NONO overexpression enhances CRC cell migration and invasion. The wound-healing assay was performed using KM12 (8 × 10^4^) or HCT8 (1 × 10^5^) cells (**g**, **i**). The transwell assay was performed using KM12 (4 × 10^4^) or HCT8 (8 × 10^4^) cells for 24 h (**h**, **j**). Scale bar, 400 μm. EV empty vector (pCDH-CMV)-transfected cells, NONO-OE NONO-overexpressing cells (transfected with pCDH-CMV-Flag-NONO). *P < 0.05, **P < 0.01, ***P < 0.001.

To investigate the role of NONO in CRC progression, we examined NONO expression levels in clinical specimens and found that it was overexpressed in CRC compared to adjacent normal tissue at both the protein (Fig. 1a and Supplementary Fig. S1a) and mRNA (Fig. 1b) levels. Consistent with these observations, *NONO* transcript level was increased in CRC samples of The Cancer Genome Atlas (TCGA) (Fig. 1b). We analyzed the correlation between NONO expression in 93 CRC tissue specimens and patients’ clinicopathologic features (Fig. 1c) and found that elevated expression of NONO was correlated with poor differentiation of intestinal tissue (*P* = 0.002; Supplementary Table S1). Kaplan–Meier survival analysis revealed that NONO overexpression was correlated with shorter overall survival (*P* = 0.012; Fig. 1d).

NONO was highly expressed in different CRC cell lines irrespective of *KRAS* mutation status (Supplementary Fig. S1b). We used KM12 and HCT8 cells—which harbor WT and mutant *KRAS*, respectively—to investigate the function of NONO in CRC. NONO knockout (KO) in both cell lines significantly inhibited proliferation, whereas NONO overexpression had the opposite effect (Fig. 1e and Supplementary Fig. S1c, d). Consistent with these results, KM12 and HCT8 NONO KO cell-derived tumors in an in vivo xenograft model were smaller than those derived from NONO WT cells (Fig. 1f and Supplementary Fig. S1e). Furthermore, the wound-healing and transwell assays showed that NONO deficiency suppressed CRC cell migration and invasion (Fig. 1g, h), while NONO overexpression enhanced these behaviors in KM12 and HCT8 cells (Fig. 1i, j).

### PRMT1 mediates NONO arginine methylation in CRC cells

**Fig. 2:**
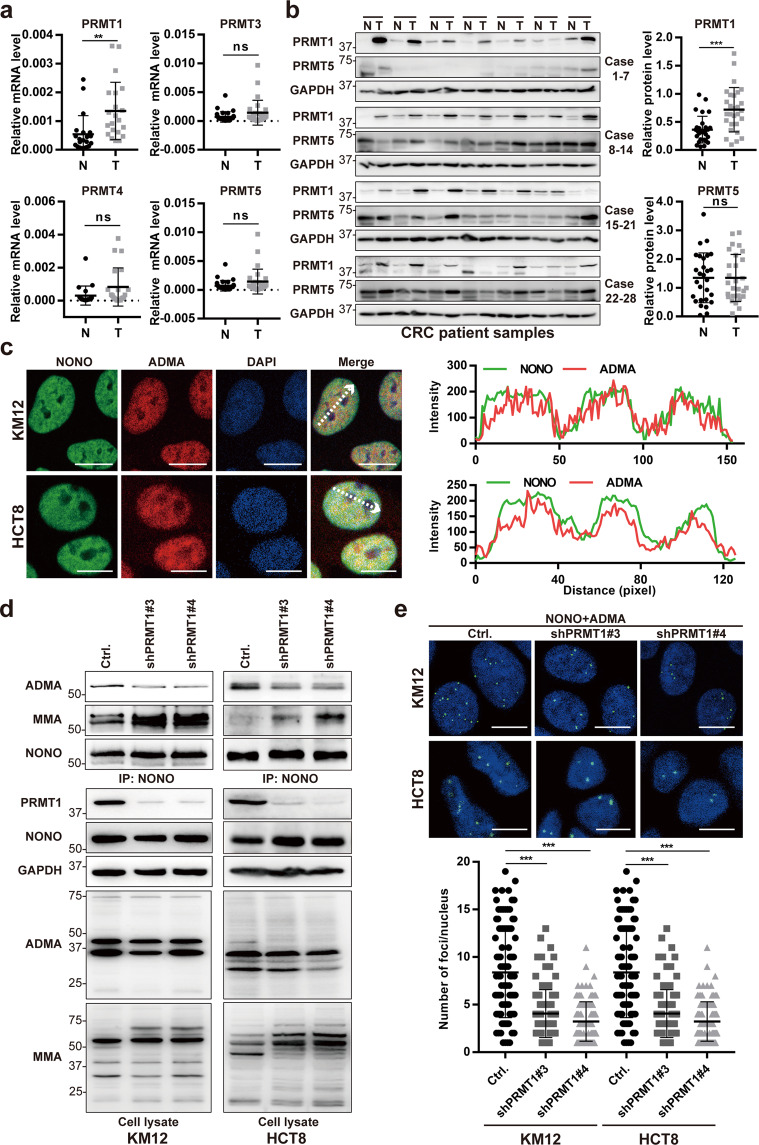
PRMT1 mediates NONO arginine methylation in CRC cells. **a** Compared to adjacent normal (N) tissue, *PRMT1* mRNA level was higher in CRC tissue (T), as determined by qPCR (*n* = 29 paired tissues). There were no differences in PRMT3, PRMT4, and PRMT5 mRNA levels between groups. **b** PRMT1 protein is highly expressed in CRC tissue. PRMT5 protein expression was similar between T and N tissues, as determined by western blotting (*n* = 28 paired tissues; left panel). Protein band intensity was quantified using ImageJ software (right panel). **c** NONO and the aDMA modification colocalized in the nucleus. Immunofluorescence analysis was performed using anti-NONO and -ADMA antibodies (right panel) in KM12 and HCT8 cells, and colocalization was analyzed using ZEN v3.0 software (Zeiss, Oberkochen, Germany) (left panel). Scale bar, 10 μm. **d**, **e** *PRMT1* silencing reduces NONO aDMA level. Endogenous NONO was immunoprecipitated (IP) from control and *PRMT1*-deficient KM12 and HCT8 cells and analyzed by western blotting (**d**). NONO methylation was detected using an anti-ADMA antibody. For Duolink PLA (**e**), NONO aDMA level was detected using anti-NONO and -ADMA antibodies (left panel). At least 100 nuclei were counted in each group (right panel). **P < 0.01, ***P < 0.001; ns no significance.

To investigate the mechanism by which NONO enhances CRC progression, Flag-NONO recombinant protein was immunoprecipitated from KM12 cells and subjected to liquid chromatography–tandem mass spectrometry (LC–MS/MS) analysis (Supplementary Fig. S2a). Several arginine residues in NONO were found to be methylated (Supplementary Table S2). To identify the PRMT responsible for arginine methylation of NONO in CRC cells, we examined PRMT1, PRMT3, PRMT4, PRMT5, PRMT6, and PRMT8 mRNA and protein levels in paired tumor and adjacent normal tissues. PRMT1 was overexpressed in CRC tissue compared to normal tissue (Fig. 2a, b). In contrast, PRMT3, PRMT4, and PRMT5 levels were comparable between groups, whereas PRMT6 and PRMT8 were undetectable in CRC tissue specimens (Fig. 2a, b). Immunofluorescence analysis and the Duolink proximity ligation assay (PLA) showed that NONO was asymmetrically dimethylated in KM12 and HCT8 CRC cells (Fig. 2c and Supplementary Fig. S2b). To determine whether asymmetric dimethylation of NONO is mediated by PRMT1, endogenous NONO was immunoprecipitated from control or *PRMT1*-silenced CRC cells, followed by immunoblotting with pan-ADMA and -MMA antibodies. Compared to control cells, NONO aDMA level was significantly decreased in *PRMT1*-deficient cells, while NONO and whole-cell lysate MMA levels were increased (Fig. 2d and Supplementary Fig. S2c). This finding was confirmed with the Duolink PLA in which NONO aDMA level was reduced in cells lacking *PRMT1* (Fig. 2e). Notably, *PRMT1* silencing had no effect on *PRMT3, PRMT4*, and *PRMT5* mRNA levels in KM12 and HCT8 cells (Supplementary Fig. S2d). These results indicate that NONO is a substrate of PRMT1 in CRC cells.

### NONO interacts with PRMT1 in CRC cells

**Fig. 3:**
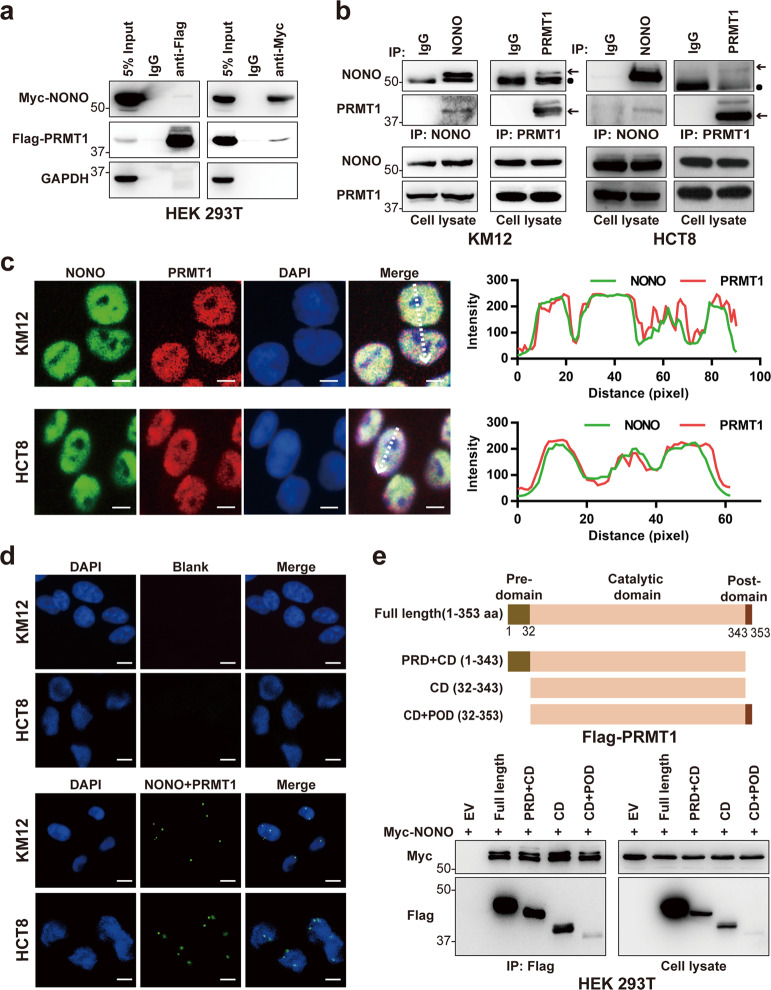
NONO interacts with PRMT1 in CRC cells. **a** Myc-NONO and Flag-PRMT1 interact in HEK 293T cells. pCDH-CMV-Myc-NONO and pCDH-CMV-Flag-PRMT1 were cotransfected into HEK 293T cells for 24 h before coIP analysis. **b** NONO interacts with PRMT1 in situ. CoIP was performed in KM12 and HCT8 cells using anti-NONO and -PRMT1 antibodies. Arrows indicate NONO or PRMT1, and circles indicate the heavy chain. **c** NONO and PRMT1 colocalize in the nucleus. Immunofluorescence analysis was performed using anti-NONO and -PRMT1 antibodies in KM12 and HCT8 cells (right panel). NONO and PRMT1 colocalization was analyzed using ZEN v3.0 software (left panel). Scale bar, 10 μm. **d** NONO interacts with PRMT1. Duolink PLA was performed with indicated antibodies. Blank, no primary antibody added in Duolink PLA. Scale bar, 10 μm. **e** NONO binds to the catalytic domain of PRMT1. NONO truncations are illustrated schematically at the top. HEK 293T cells were cotransfected with Myc-NONO and Flag-PRMT1 truncations for 24 h before CoIP analysis. aa amino acid, CD catalytic domain, POD post domain, PRD pre domain.

To confirm whether NONO interacts with PRMT1, we performed coimmunoprecipitation (coIP) assays. PRMT1 was detected in Myc-NONO complexes and NONO was present in Flag-PRMT1 complexes immunoprecipitated from HEK 293T cells (Fig. 3a). The coIP assay confirmed the interaction between NONO and PRMT1 in KM12 and HCT8 cells (Fig. 3b). Immunofluorescence analysis and the Duolink PLA showed that NONO and PRMT1 colocalized and interacted in the nucleus (Fig. 3c, d). A coIP experiment with NONO and various truncation mutants of PRMT1 showed that the catalytic domain of PRMT1 mediates its interaction with NONO (Fig. 3e).

### PRMT1-mediated methylation at R251 is required for the oncogenic function of NONO

**Fig. 4:**
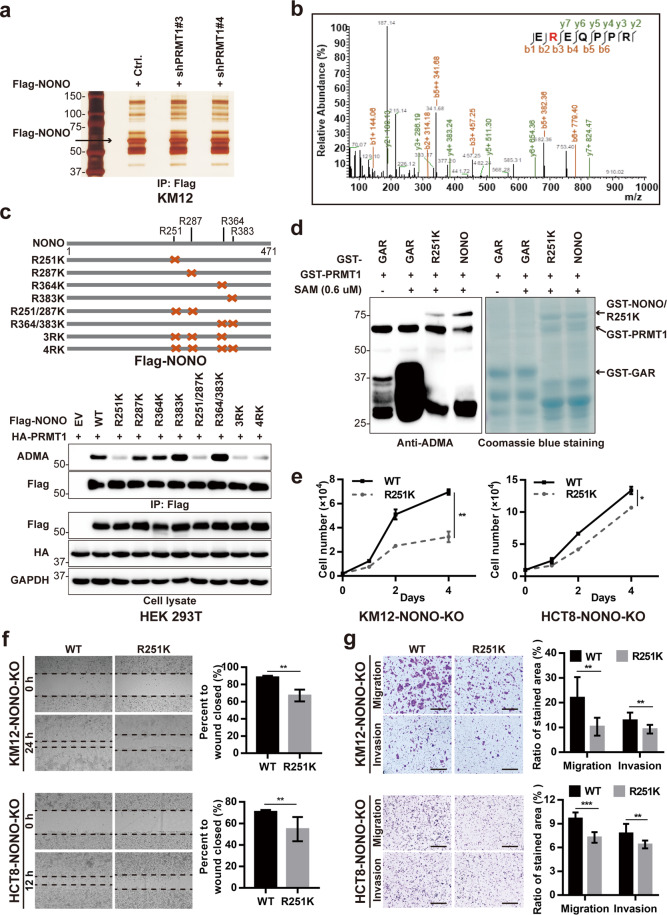
PRMT1-mediated methylation at R251 is required for the oncogenic function of NONO. **a** Flag-NONO protein were immunoprecipitated from control or *PRMT1*-silenced KM12 cells, separated by SDS–PAGE, and subjected to silver staining. **b** LC–MS/MS analysis of the NONO R251 methylation site. The fragmentation pattern of the typical NONO peptide EREQPPR is shown. Fragment ions are shown as b and y ions; ++ represents the loss of doubly charged ions. **c** R251K mutation abolishes PRMT1-mediated aDMA modification of NONO. NONO R-to-K mutants are illustrated schematically at the top. WT or R-to-K mutant NONO protein was immunoprecipitated with Flag beads and then immunoblotted with anti-ADMA antibody. **d** NONO is methylated by PRMT1 at R251, as determined with the in vitro methylation assay. GST-tagged GAR, NONO, and R251K were incubated with purified GST-PRMT1 in the presence or absence of 0.6 μM SAM. Proteins were separated on SDS–PAGE and subjected to western blotting analysis and Coomassie blue staining. GAR, glycine- and arginine-rich N-terminal region of fibrillarin. **e** NONO R251K mutation reduces cell proliferation. KM12 (1 × 10^4^) and HCT8 (2 × 10^4^) cells were seeded on day 0 and counted on days 2 and 4. NONO R251K mutation inhibited KM12 cell migration in the wound-healing assay (**f**) and invasion in the transwell assay (**g**). For experiments shown in **e**–**g**, KM12 cells were transfected with pCDH-CMV-Flag-NONO (WT) or pCDH-CMV-Flag-NONO-R251K (R251K mutant) for 24 h before the indicated assay. Scale bar, 400 μm. *P < 0.05, **P < 0.01, ***P < 0.001.

To identify the arginine residues of NONO that are methylated by PRMT1, Flag-NONO was immunoprecipitated from control and *PRMT1*-silenced KM12 cells and subjected to LC–MS/MS analysis (Fig. 4a). Compared to control cells, Flag-NONO protein from *PRMT1*-silenced cells showed decreased methylation at R251, R287, R364, and R383, although only R251 showed a statistically significant difference in methylation level (*P* < 0.05; Fig. 4b, Supplementary Fig. S3a, and Supplementary Table S2). To confirm these findings, a series of R-to-K NONO mutants and PRMT1 were coexpressed in HEK 293T cells, followed by analysis of aDMA status. aDMA was not detected in R251K mutants but was observed in R287K, R364K, and R383K mutants (Fig. 4c). After in vitro incubation with PRMT1, purified NONO protein showed an increase in asymmetric arginine dimethylation, whereas the R251K mutant had a lower aDMA level (Fig. 4d).

R251 methylation had no influence on NONO expression (Supplementary Fig. S3b), but we investigated it’s impact on the oncogenic function of NONO. The results of the cell proliferation, wound-healing, and transwell assays revealed that compared to WT NONO, CRC cells expressing hypomethylated NONO (R251K) showed reduced growth, migration, and invasion (Fig. 4e–g). Thus, PRMT1-mediated R251 methylation promotes the oncogenic function of NONO in CRC.

### PRMT1 has an oncogenic function that is correlated with poor outcome in CRC

**Fig. 5:**
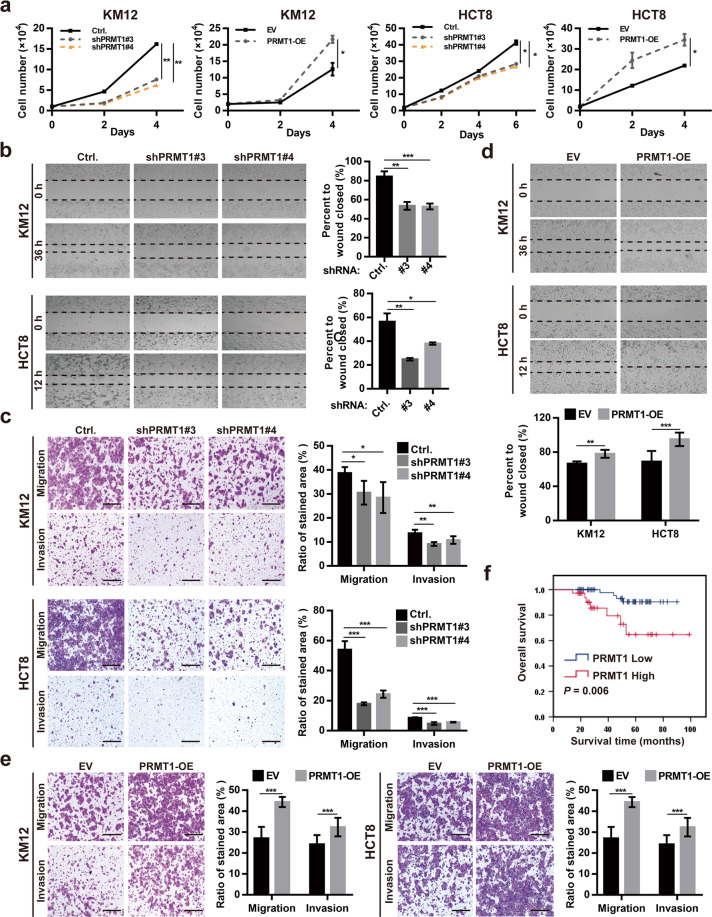
PRMT1 has an oncogenic function that is correlated with poor outcome in CRC. **a** PRMT1 promotes CRC cell proliferation. KM12 (1 × 10^4^) and HCT8 (1.8 × 10^4^ or 2 × 10^4^) cells were seeded on day 0 and counted every 2 days. *PRMT1* knockdown inhibited and *PRMT1* overexpression promoted, KM12 cell migration in the wound-healing assay (**b**, **d**) and invasion in the transwell assay (**c**, **e**). KM12 cells were transfected with pCDH-CMV-Flag-PRMT1 (PRMT1-OE) or empty vector (EV) for 24 h before the indicated assay. EV empty vector (pCDH-CMV) transfected cells, NONO-OE NONO-overexpressing cells (transfected with pCDH-CMV-Flag-NONO). Scale bar, 400 μm. **f** Elevated PRMT1 expression is correlated with shorter overall survival in CRC patients. The 97 patients were divided into PRMT1 low (*n* = 61) and high (*n* = 36) expression subgroups. *P < 0.05, **P < 0.01, ***P < 0.001.

Given that PRMT1 was overexpressed in CRC tissue and PRMT1-mediated arginine methylation of NONO enhanced tumor growth and metastasis, we hypothesized that tumor progression induced by NONO arginine methylation was initiated by PRMT1. *PRMT1* knockdown inhibited cell proliferation, whereas its overexpression had the opposite effect (Fig. 5a). The wound-healing and transwell assays showed that PRMT1 silencing suppressed KM12 and HCT8 cell migration and invasion (Fig. 5b, c). Conversely, PRMT1 overexpression enhanced these malignant behaviors (Fig. 5d, e). *PRMT1* transcript level was higher in CRC than in normal tissue in TCGA (Supplementary Fig. S4a). Immunohistochemical (IHC) analysis of CRC tissue confirmed that PRMT1 was highly expressed in the CRC zone (Supplementary Fig. S4b). Moreover, elevated PRMT1 expression was correlated with advanced TNM stage (*P* = 0.040; Supplementary Table S3) and shorter overall survival (*P* = 0.006; Fig. 5f) in CRC patients.

### PRMT1 inhibition reduces NONO arginine methylation and suppresses CRC progression

**Fig. 6:**
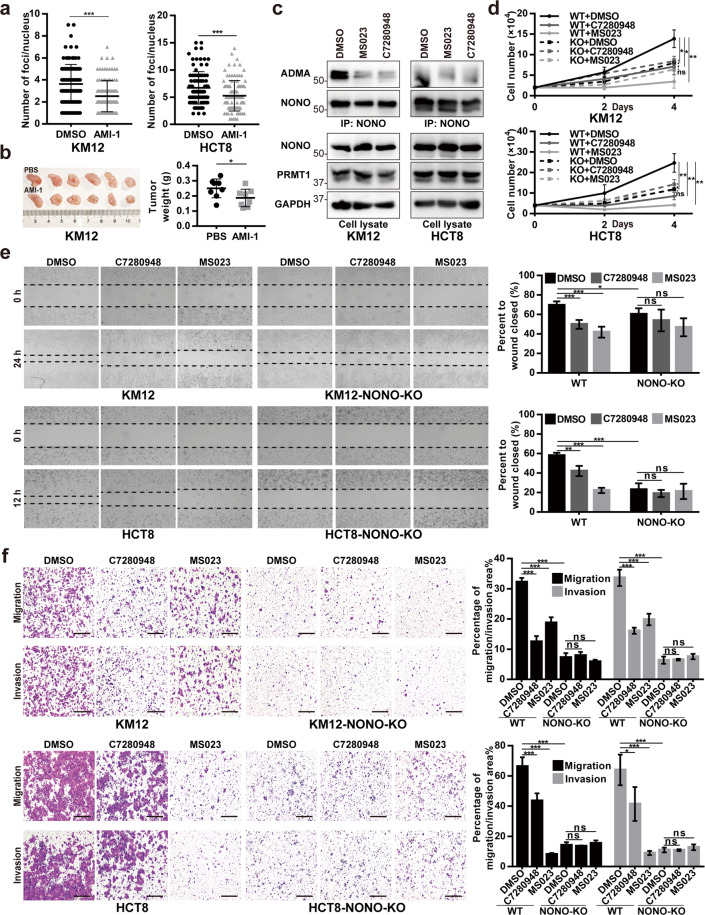
PRMT1 inhibition reduces NONO arginine methylation and suppresses CRC progression. **a** Treatment with the PRMT1 inhibitor AMI-1 reduced NONO aDMA level. KM12 and HCT8 cells treated with AMI-1 (1.2 and 0.6 mM) or DMSO for 48 h were subjected to Duolink PLA with anti-ADMA antibody. **b** AMI-1 treatment reduced tumor xenograft weight (*n* = 6 mice per group). **c** Treatment with the PRMT1 inhibitors MS023 and C7280948 decrease aDMA NONO level. KM12 and HCT8 cells were treated with 10 μM MS023 and 40 μM C7280948, respectively, for 48 h, and subjected to NONO IP and western blotting analysis with anti-ADMA antibody. **d** Treatment with MS023 or C7280948 inhibits CRC cell proliferation. KM12 WT/NONO-KO (2 × 10^4^) and HCT8 WT/NONO-KO (4 × 10^4^) cells were seeded on day 0 and counted on days 2 and 4 after treatment with the indicated inhibitor. KM12 and HCT8 WT/NONO-KO cells treated with MS023 and C7280948 were subjected to the wound-healing assay (**e**) and transwell assay (**f**), respectively. For (**d**–**f**), MS023, C7280948, or DMSO was added at the time the cells were seeded. Scale bar, 400 μm. *P < 0.05, **P < 0.01, ***P < 0.001.

To further confirm the role of PRMT1 in promoting CRC progression, we used the PRMT1-selective small-molecule inhibitors AMI-1, MS023, and C7280948 to prevent substrate recognition or binding by PRMT1 [28–30]. The Duolink PLA showed that NONO aDMA level was significantly reduced in AMI-1–treated KM12 and HCT8 CRC cells compared to control cells (Fig. 6a and Supplementary Fig. S4c) and in the xenograft model, mice treated with AMI-1 had smaller tumors (Fig. 6b). MS023 and C7280948 also reduced NONO aDMA level in KM12 and HCT8 cells, as demonstrated with western blotting assay (Fig. 6c), and suppressed cell proliferation, migration, and invasion (Fig. 6d–f). However, these effects were not observed in NONO-depleted KM12 and HCT8 cells treated with the inhibitors (Fig. 6d–f), indicating that NONO mediates the oncogenic effects of PRMT1 in CRC. Taken together, these findings suggest that inhibiting NONO arginine methylation by PRMT1 can prevent the malignant transformation of CRC irrespective of *KRAS* mutation status.

## Discussion

**Fig. 7:**
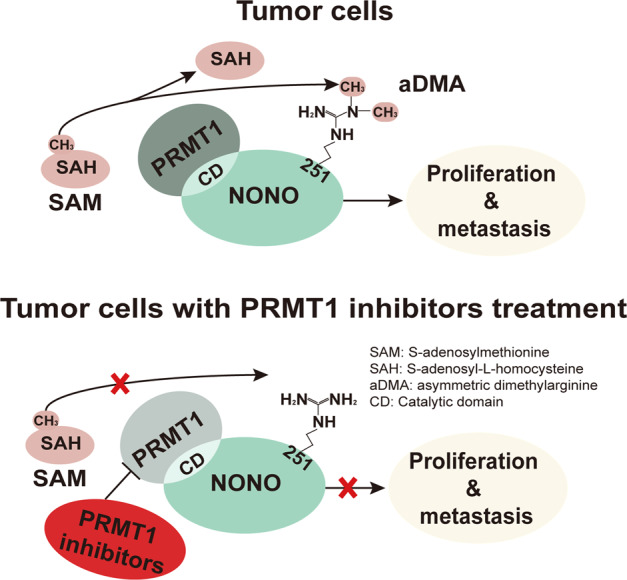
Model of PRMT1-mediated NONO arginine methylation in CRC progression. In CRC cells, PRMT1-mediated methylation of NONO at R251 promotes tumor cell proliferation and metastasis. Malignant progression induced by NONO arginine methylation may be abrogated by treatment with small-molecule inhibitors of PRMT1 (AMI-1, MS023, and C7280948).

Anti-EGFR/vascular endothelial growth factor receptor-based targeted therapy as well as immunotherapy involving programmed death (PD)−1/programmed death ligand (PD-L)1 blockade have shown survival benefits in advanced CRC patients with metastasis [31]. Clarifying the molecular mechanisms of metastasis can reveal novel therapeutic targets. In this study we found that NONO was overexpressed and arginine-methylated in CRC tissue, regardless of *KRAS* mutation status. We demonstrated that the R251 residue of NONO was asymmetrically dimethylated by PRMT1 to promote the proliferation, migration, and invasion of CRC cells. Aberrant expression of PRMT1 and NONO was correlated with shorter overall survival in CRC patients. Suppressing PRMT1 by knocking down its expression or using pharmacological inhibitors abrogated the increases in CRC cell proliferation, migration, and invasion mediated by R251-methylated NONO, an effect that was independent of *KRAS* mutation status (Fig. 7).

Aberrant expression of NONO is correlated with malignant phenotypes in various cancers [32, 33]. NONO increases lncRNA Prostate cancer gene expression marker (PCGEM)1 expression via upregulation of androgen receptor [34], resulting in castration-resistant prostate cancer [35]. The NONO/PSF complex is a critical regulator of radiation-induced DNA double-stranded breaks or ultraviolet-induced DNA damage and confers resistance to radiotherapy in melanoma cells [36]. Knockdown of NONO/RALY complex components reversed YB-1 overexpression-induced oxaliplatin resistance in CRC cells [37], while NONO silencing modulated the cellular response to ultraviolet-induced DNA damage in melanoma cells [38]. However, NONO is a nuclear protein that cannot be directly targeted by currently available antibody drugs or small-molecule inhibitor compounds. Therefore, other related molecules are alternative therapeutic targets for CRC treatment. We showed here that PRMT1-mediated arginine methylation is essential for the oncogenic function of NONO, and that expressing the R251K arginine methylation mutant of NONO in CRC cells attenuated their malignant behaviors. Similar effects were observed in vitro and in vivo by blocking NONO arginine methylation with the PRMT1-specific inhibitors AMI-1, MS023, and C7280948. Thus, therapeutic targeting of PRMT1-mediated arginine methylation is a promising strategy for CRC treatment.

R251K mutation largely abolished arginine methylation of NONO by PRMT1, indicating that the R251 residue is critical for the oncogenic function of NONO in CRC. Pharmacological inhibition of PRMT1 blocked NONO arginine methylation and abrogated the oncogenic phenotypes associated with aDMA NONO. Interestingly, R251 is not located in the glycine- and arginine-rich motif, a consensus sequence for PRMTs [23]. Several proteins including signal transducer and activator of transcription (STAT)1 [39], peroxisome proliferator-activated receptor gamma coactivator (PGC)−1α [40], and runt-related transcription factor (RUNX)1 [41] are methylated by PRMTs at nonconsensus sites; meanwhile, other proteins containing a GAR motif such as TR3 interact with but are not a substrate of PRMT1 [42], suggesting that the motif is not be essential for PRMT1-mediated arginine methylation.

We previously reported that PRMTs may share substrates and have distinct roles in different tumor types. In CRC, methylation of R198/200 in the extracellular domain of EGFR by PRMT1 enhanced receptor dimerization and cell proliferation, resulting in tumor cell resistance to the anti-EGFR monoclonal antibody cetuximab [26]. However, in breast cancer cells, abolishing PRMT5-mediated EGFR Arg1175 methylation increased EGF-induced extracellular signal-regulated kinase (ERK) activation by preventing the recruitment of Src homology 2 domain-containing protein tyrosine phosphatase (SHP)1 to EGFR, leading to enhanced cell proliferation, migration, and invasion of EGFR-overexpressing tumor cells [43]. In the present work, we found that PRMT1 but not other PRMTs was overexpressed in CRC tissue relative to adjacent normal tissue, and catalyzed arginine methylation of NONO. PRMT3 and PRMT4 also catalyzed arginine methylation of NONO, but at residues other than R251 (Supplementary Fig. S5). A previous study reported that NONO was methylated by PRMT4 at R357, R365, and R378 [19]. Therefore, NONO R251 may be an arginine residue specifically methylated by PRMT1. Notably, *PRMT1* knockdown enhanced the antitumor efficacy of cetuximab in both *KRAS* mutant and WT CRC cells, suggesting that targeting PRMT1 may sensitize CRC cells to cetuximab regardless of *KRAS* mutation status [26]. We also found that PRMT1 enhanced CRC cell proliferation and metastasis via an EGFR-independent mechanism of asymmetric dimethylation of NONO at R251 that was independent of the presence of *KRAS* mutation, as the effect was observed in both KM12 cells expressing WT *KRAS* and *KRAS-*mutant HCT8 cells.

Arginine methylation regulates signal transduction cascades [44]. In order to elucidate the mechanism underlying the arginine methylation-mediated oncogenic function of NONO, we carried out RNA sequencing analysis of CRC cells expressing WT and R251K-mutant NONO. The results indicated that the cGMP-dependent protein kinase or protein kinase G and cAMP pathways are potential targets of arginine-methylated NONO (Supplementary Fig. S6a–c). NONO is required for cAMP-dependent activation of CREB target genes [45], and NONO-mediated cAMP signaling is involved in tumor growth [15, 46] or metastasis [14]. In this context, our current findings imply that aDMA R251 is responsible for the tumor-enhancing effects of NONO in CRC, including increased cell migration and invasion, which are independent of classic epithelial-to-mesenchymal transition signaling pathways (Supplementary Fig. S6d, e) but may be mediated by cAMP signaling.

In summary, we demonstrated that PRMT1-mediated asymmetric dimethylation of NONO at R251 promotes CRC progression by enhancing tumor cell proliferation, migration, and invasion. These results imply that small-molecule inhibitors of PRMT1 have clinical potential for the treatment of advanced CRC.

## Materials and methods

More details are provided in the Supporting Information.

### Tissue specimens

Four sets of human CRC samples were used in this study. NONO, PRMT1, PRMT3, PRMT4, PRMT5, PRMT6, and PRMT8 mRNA and protein expression in tumor and adjacent normal tissues was evaluated in cohort I (28 CRC patients) and cohort II (29 CRC patients), respectively, by quantitative (q)PCR and western blotting, respectively. Tumor specimens from cohort III (93 locally advanced CRC patients) and cohort IV (97 locally advanced CRC patients) were used to analyze the correlation between clinical outcome and NONO and PRMT1 expression detected by immunohistochemistry. Locally advanced CRC was defined as T3–4/N + M0 (stage II/III) according to the American Joint Committee on Cancer TNM Classification System (8th Edition) [47], and was diagnosed based on retrospective review of contrast-enhanced pelvic magnetic resonance imaging. CRC patients with distant metastasis were excluded. All cases received standard treatment of neoadjuvant chemoradiotherapy followed by radical surgery and adjuvant chemotherapy. Tissue samples were obtained from the Tissue Bank of the Sixth Affiliated Hospital of Sun Yat-sen University with approval from the Human Medical Ethics Committee of Sun Yat-sen University and with written informed consent from all patients for the use of their data. Clinicopathologic parameters and follow-up information were obtained from the Follow-up Database of the Sixth Affiliated Hospital of Sun Yat-sen University.

### Plasmid construction and lentiviral infection

Human *Flag-NONO, Myc-NONO, HA-PRMT1, Flag-PRMT1* and its truncated mutants (1–343 aa, 32–343 aa, and 32–353 aa) were cloned into pCDH-CMV for transient transfection. Using the pCDH-CMV-Flag-NONO as a template, *Flag-NONO* R-to-K mutants (R251K, R287K, R364K, R383K, R251/287K, R364/383K, 3RK, 4RK) were developed using mutagenesis kit (cat. no. SMK-101; Toyobo, Osaka, Japan). Human *PRMT1*, *NONO*, *NONO-R251K*, and GAR were cloned into pGEX-6P-1 for protein purification.

To knockout endogenous *NONO*, LentiCRISPR-Dual-sgNONO plasmid was constructed by inserting a fragment containing sgRNA#1-scaRNA-pH1 (H1 promoter)-sgRNA#2 into lentiCRISPR vector (cat. no. 98290; Addgene, Watertown, MA, USA). To knockdown endogenous *PRMT1*, pLKO.1-shPRMT1 were constructed by inserting the shRNA of *PRMT1* into pLKO.1 (cat. no. 8453; Addgene) according to the Addgene website protocol (http://www.addgene.org/protocols/plko/).

For virus production and infection, plasmids were cotransfected with psPAX2 (cat. no. 12260; Addgene) and pMD2.G (cat. no. 12259; Addgene) into HEK 293T cells for 48 h. The culture medium was collected, supplemented with polybrene (10 μg/ml) and incubated with KM12 and HCT8 cells for 48 h. Cells were selected with puromycin (2 μg/ml) to generate stable cell lines.

The sequences of all primers were listed in Supplementary Table S4.

### RNA extraction and qPCR

Total RNA was isolated from tissues or cells using TRIzol reagent (Invitrogen, Thermo Fisher Scientific, Waltham, MA, USA) according to manufacturer’s instructions, and reverse-transcribed to cDNA using qPCR RT Master Mix (cat. no. FSQ-301; Takara, Shiga, Japan). qPCR analyses were performed using SYBR Green (cat. no. QPK-201; Takara) on a LightCycler 480 instrument (Roche, Basel, Switzerland). Gene expression was normalized to *U6* for tissues and *GAPDH* for cells. The primer sequences used in the present study were listed in Supplementary Table S4.

### Western blotting assay

Western blotting assay was performed as described previously [38]. The following antibodies were used: Flag (cat. no. ab49763; abcam, Cambridge, MA, USA), HA (cat. no. ab1265; abcam), Myc (cat. no. ab9106; abcam), GAPDH (cat. no. 60004–1-Ig; Proteintech, Chicago, IL, USA), NONO (cat. no. 611279; BD Bioscience, Franklin lakes, NJ, USA), PRMT1 (cat. no. 2449; Cell Signaling Technology, CST, Beverly, MA, USA), PRMT5 (cat. no. D160716; Sangon Biotech, Shanghai, China), pan-ADMA (cat. no. 13522; CST), pan-MMA (cat. no. 8015; CST). Images were captured with ChemiDocTM Imaging System (Bio-Rad, Hercules, CA, USA), and the band intensity were analyzed using Image J software (National Institutes of Health, Bethesda, MD, USA).

### IHC staining

IHC for NONO (1:1500; cat. no. 611279; BD Bioscience) and PRMT1 (1:1000; cat. no. 11279–1-AP; Proteintech) were performed on CRC tissue. The IHC score ranging from 0 to 3 according to the ToGA trial [48] was used to determine the intensity and the percentage of stained cancer cells. According to IHC score, sections were ranked into two groups: low expression (*H* score was 0 and 1) and high expression (*H* score was 2 and 3).

### Cell counting, migration, and invasion assays

Cell counting assay was used to analyze the proliferation of cells. KM12 (1~2 × 10^4^) or HCT8 (1.8~4 × 10^4^) cells were seeded in 24-well plates with triplicate. Cell numbers were counted every two days.

Wound-healing assay was used to examine the migration capacity of tumor cells in vitro. KM12 (8 × 10^4^) or HCT8 (1 × 10^5^) cells were cultured in 2 well culture-inserts (cat. no. 80209; iBidi, Martin Reid, Germany) overnight for wound generating. The wounds were captured every 12 h with microscope (Olympus, Tokyo, Japan) and were measured by Adobe Photoshop CC 2017 (San Jose, CA, USA).

Transwell assay was performed to analyze the migration and invasive capacity of tumor cells in Cell Culture Inserts of 24 well with 8.0 μm pore size (Falcon, BD Bioscience). For migration assay, KM12 (4 × 10^4^) or HCT8 (8 × 10^4^) cells in 100 μl serum-free medium were seeded in the top chamber. Medium containing 10% FBS was used as a chemoattractant in the lower chamber. For inhibitor treatment, 0.6–1.2 mM AMI-1 (cat. no. 7884), 10 μM MS023 (S8112), 40 μM C7280948 (cat. no. S6737; all inhibitors form Selleck chemicals, Houston, Texas, USA) or equal volume of Dimethyl sulfoxide (DMSO, cat. no. D8418; Sigma-Aldrich, St. Louis, MO, USA) was added into the top chamber. After incubation for 24 h, cells were fixed with 4% paraformaldehyde and stained with 0.1% crystal violet (cat. no. 0424A17; Leagene, Beijing, China). Cells were captured in five random fields with microscope (Olympus). For invasion assay, the membranes of inserts were coated with Matrigel (1:20, cat. no. 356234; Corning, NY, USA) to form matrix barriers before seeding cells.

### Duolink PLA

aDMA NONO and the interaction between NONO and PRMT1 were detected using Duolink PLA kits (cat. no. DUO92014 and DUO92008; Sigma-Aldrich) according to the manufacturer’s instructions. The primary antibodies used for the assay were the same as those used for immunofluorescence analysis. KM12 cells (4 × 10^4^) or HCT8 cells (8 × 10^4^) were seeded on slides and cultured for 48 h. After fixation and blocking, the slides were incubated with primary antibodies for 2 h at room temperature, followed by PLUS (cat. no. DUO92001) and MINUS (cat. no. DUO92005) PLA probes (both from Sigma-Aldrich) for 1 h at 37 °C. The slides were incubated in ligation solution for 30 min at 37 °C and in amplification solution for 100 min at 37 °C. After nuclear staining with 4′,6-diamidino-2-phenylindole, fluorescent foci representing methylated NONO or NONO/PRMT1 complex were visualized and imaged with a confocal microscope (model TCS-SP8; Leica, Wetzlar, Germany).

### CoIP assay

Cells were resuspended in radioimmunoprecipitation assay (RIPA) lysis buffer (150 mM NaCl, 50 mM Tris-HCl [pH 7.4], 1% Nonidet P-40, and 1 mM EDTA) with protease and phosphatase inhibitor cocktails (cat. no. 04693132001 and 04906837001, respectively; Roche, Basel, Switzerland). After incubation for 30 min on ice, the cells were centrifuged at 13,000 *g* for 15 min at 4 °C to remove cellular debris. Proteins in the whole-cell lysate were quantified with a bicinchoninic acid assay kit (cat. no. 23225; Thermo Fisher Scientific).

For immunoprecipitation (IP) of Flag- or Myc-tagged proteins, cleared lysate was mixed with anti-Flag magnetic beads (cat. no. M8823; Sigma-Aldrich) or anti-Myc magnetic beads (cat. no. B26301; Bimake, Houston, TX, USA) for 2–4 h at 4 °C. For IP of endogenous NONO or PRMT1 protein, cleared lysate was incubated overnight at 4 °C with 0.5–2 μg of anti-NONO antibody (cat. no. 611279; BD Bioscience), anti-PRMT1 antibody (cat. no. 2449; CST), or isotype-matched IgG. Dynabeads Protein G (Invitrogen, Carlsbad, CA, USA) were then added, and the tube was rotated for 2–4 h at 4 °C. The beads were washed three times with RIPA buffer and incubated with 0.1 M glycine HCl (pH 3.1) to release immunoprecipitated proteins. The supernatant was neutralized with 1 M NaOH and analyzed by western blotting.

### In vitro methylation assay

Substrate protein (purified GST-GAR, GST-NONO, or GST-NONO-R251K; 10 μg) was incubated with 1 μg of GST-PRMT1 and 0.6 mM nonradioactive SAM (cat. no. B9003S; New England Biolabs, Ipswich, MA, USA) in 30 μl of methyltransferase reaction buffer (20 mM Tris-HCl [pH 8.0], 200 mM NaCl, and 0.4 mM EDTA) for 1 h at 37 °C. The reaction was terminated by adding 5× SDS loading buffer and heating for 10 min at 100 °C. Samples were divided into two equal parts: one was used for detection of the methylation signal by western blotting, and the other was subjected to Coomassie blue staining as the loading control.

### Mouse xenograft model

Female BALB/c nude mice (5 weeks old) were purchased from GemPharmatech (Jiangsu, China) and maintained in a specific pathogen-free room on a 12:12-h light/dark cycle, and were fed autoclaved chow and water. KM12/HCT8 WT or NONO KO cells (1 × 10^6^) were resuspended in 100 μl of phosphate-buffered saline (PBS) and then subcutaneously injected into the right or left posterior flank of each mouse. The animals were sacrificed 3 weeks later and tumors were excised and weighted.

For AMI-1 treatment, 2 weeks after implantation, AMI-1 (0.5 mg in 100 μl PBS) or 100 μl PBS was intratumorally injected once a day for 7 days [49]. On day 8, animals were sacrificed and the tumors were weighted. Experiments involving animals were used randomly and approved by the Institutional Animal Care and Use Committee of the Sixth Affiliated Hospital of Sun Yat-sen University.

### Statistical analysis

All data are shown as mean ± standard deviation of at least three independent experiments. Differences between two subgroups were analyzed with the unpaired *t* test using Prism v7.0 software (GraphPad Inc, San Diego, CA, USA). Differences in NONO, PRMT1, PRMT3, PRMT4, and PRMT5 mRNA and protein levels between paired tumor and adjacent normal tissues were compared with the paired *t* test using Prism v7.0. Overall survival was analyzed with the Kaplan–Meier method and the log-rank test using SPSS v25.0 software (SPSS Inc, Chicago, IL, USA). *P* < 0.05 was considered significant.

## Supplementary information

Supplementary materials
