# Supplementary material for: PRMT1 enhances oncogenic arginine methylation of NONO in colorectal cancer
Source: Oncogene. 2021 Jan 8;40(7):1375–89. doi: 10.1038/s41388-020-01617-0 (PMC7892343; doi:10.1038/s41388-020-01617-0)
Supplement: Supplementary file 1 — Supplementary materials [file 41388_2020_1617_MOESM1_ESM.pdf]

PRMT1 enhances oncogenic arginine methylation of NONO in colorectal cancer

Xin-Ke Yin, Yun-Long Wang, Fei Wang, Wei-Xing Feng, Shao-Mei Bai, Wan-Wen Zhao, Li-Li Feng, Ming-Biao Wei, Cao-Litao Qin, Fang Wang, Zhi-Li Chen, Hong-Jun Yi, Yan Huang, Pei-Yi Xie, Taewan Kim, Ying-Nai Wang, Jun-Wei Hou, Chia-Wei Li, Quentin Liu, Xin-Juan Fan, Mien-Chie Hung, Xiang-Bo Wan

Inventory of supplementary data

1. Supplementary Materials and Methods……………………Page 2-5
2. Supplementary Figures and legends……………………….Page 6-11
3. Supplementary Tables……………………………………..Page 12-17

Supplementary Materials and Methods

Cell lines and cell culture

Human KRAS WT (Caco2, HT29, Colo205, KM12 and RKO) and mutant (SW480, SW620, T84, HCT116, HCT15, HCT8, LS174T and DLD1) CRC cell lines were cultured in RPMI 1640 (Gibco; Waltham, MA, USA) supplemented with 10% FBS (Gibco). HEK 293T cells were cultured in DMEM (Gibco) supplemented with 10% FBS. For Caco2, T84 and KM12 cell lines were obtained from the Cell Bank of Type Culture Collection of Chinese Academy of Sciences (Shanghai, China), and the other cell lines were obtained from the American Type Culture Collection (Rockville, MD, USA). All cell lines were identified by short tandem repeat analysis performed from cell banks. All experiments were performed on KM12 and HCT8 cells between passages 3 and 10, and HEK 293T between passages 3 and 14.

LC–MS/MS analysis

Flag-NONO protein was purified from pCDH-CMV-Flag-NONO-transfected control or PRMT1-deficient KM12 cells using anti-Flag Magnetic Beads (cat. no. M8823; Sigma-Aldrich) and separated by sodium dodecyl sulfate (SDS)–PAGE. The gel was subjected to silver staining using the Pierce Silver Stain for Mass Spectrometry kit (cat. no. 24600; Thermo Fisher Scientific, Waltham, MA, USA). The Flag-NONO band was excised and incubated in destaining solution (25 mM NH4HCO3, 50% acetonitrile) for 20 min, and dried in acetonitrile. The gel was then incubated in 10 mM dithiothreitol

for 1 h at 56°C and in 55 mM iodoacetamide for 45 min at room temperature to denature the proteins. After washing with 25 mM NH4HCO3, the gel was dried in acetonitrile, and proteins were digested overnight at 37°C with 1 μg/μl trypsin solution. The reaction was terminated by adding 0.1% formic acid solution. The peptide solution was collected and concentrated to ~20 μl in a SpeedVac (Thermo Fisher Scientific). LC–MS/MS analysis was carried out on a Q-Exactive system (Thermo Fisher Scientific) at Beijing Protein Innovation (Beijing, China).

Protein purification

Plasmids containing PRMT1, NONO, NONO-R251K, or GAR (glycine- and arginine-rich N-terminal region of fibrillarin) fused to glutathione S-transferase (GST) were transformed into Escherichia coli Rosetta 2(DE3) cells (cat. no. CB105; Tiangen, Beijing, China). A single bacterial colony was inoculated into Luria–Bertani medium containing ampicillin and cultured overnight at 37°C. Protein expression was induced overnight at 16°C with 0.5 mM isopropyl β-D-1-thiogalactopyranoside (cat. no. A600168; Sangon Biotech). Cells were collected, centrifuged, and resuspended in a buffer composed of 150 mM NaCl, 2.7 mM KCl, 10 mM Na2HPO4 (pH 7.3), and 1.8 mM KH2PO4 and supplemented with 1 mM phenylmethylsulfonyl fluoride. The cells were lysed by sonication and then centrifuged at 12 000 g for 30 min at 4°C. Soluble proteins were purified using GST-tag Purification Resin (cat. no. P2253; Beyotime, Shanghai, China) and eluted in buffer composed of 50 mM Tris-HCl (pH 8.0) and 10

mM glutathione. Further concentration and buffer exchange were performed using Amicon Ultra-0.5/4 spin columns (Merck Millipore, Darmstadt, Germany). The purified protein was quantified with a BCA protein assay kit and verified by Coomassie staining.

Immunofluorescence staining

After seeding on slides (cat. no. J24001; JingAn Biological, Shanghai, China), cells were fixed with 4% paraformaldehyde, permeabilized with 0.1% Triton, and incubated with blocking solution [5% BSA (cat. no. V900933; Sigma-Aldrich) in 1×PBS]. Slides were then incubated with anti-NONO antibody (1:200; cat. no. 611279; BD Bioscience), anti-PRMT1 antibody (1:100; cat. no. 2449; CST) or/and anti-pan-ADMA antibody (1:100; cat. no. 13522; CST) for 2 h at room temperature. Slides were stained with anti-Rabbit Alexa Fluor 555 Probes (1:200; cat. no. 4413; CST), anti-Mouse Alexa Fluor 488 Probes (1:200; cat. no. 4408; CST) and DAPI (cat. no. D9542; Sigma-Aldrich) for 1 h at room temperature. The images were captured by confocal microscope (TCS-SP8, Leica, Weztlar, Germany). The co-localization between NONO, PRMT1 and ADMA were analyzed by ZEN v3.0 (blue edition, Zeiss, Jena, Germany).

Immunohistochemical (IHC) staining

IHC staining was performed to evaluate the protein expression in paraffin-embedded human tissue sections. After baked at 65 °C for 1 hour, sections were deparaffinized in

xylene and rehydrated through a series of ethanol solutions, treated with 1% hydrogen peroxide solution to block the endogenous peroxidase, and processed for antigen retrieval by boiling in citrate buffer using a microwave oven. Anti-NONO antibody (1:1500; cat. no. 611279; BD Bioscience) or anti-PRMT1 antibody (1:1000; cat. no. 11279-1-AP; Proteintech) was incubated with the sections for overnight at 4 ℃. After washing, the sections were incubated with anti-rabbit/mouse secondary antibodies (cat. no. MP-7401/4702; Vectorlabs, Burlingame, CA, USA) for 30 min at room temperature. Immunostaining was performed using 3, 3'-diaminobenzidine kit (cat. no. ZLI-9018; ZSGB-Bio, Beijing, China), which shown as a brown-colored precipitate at the antigen site. Sections were then counterstained in hematoxylin, and followed by dehydrating and mounting. The IHC score ranging from 0 to 3 according to the ToGA trial was used to determine the intensity and the percentage of stained cancer cells. According to IHC score, sections were ranked into two groups: low expression (H score was 0 and 1) and high expression (H score was 2 and 3).

Supplementary Figures and legends

Supplementary Figure S1

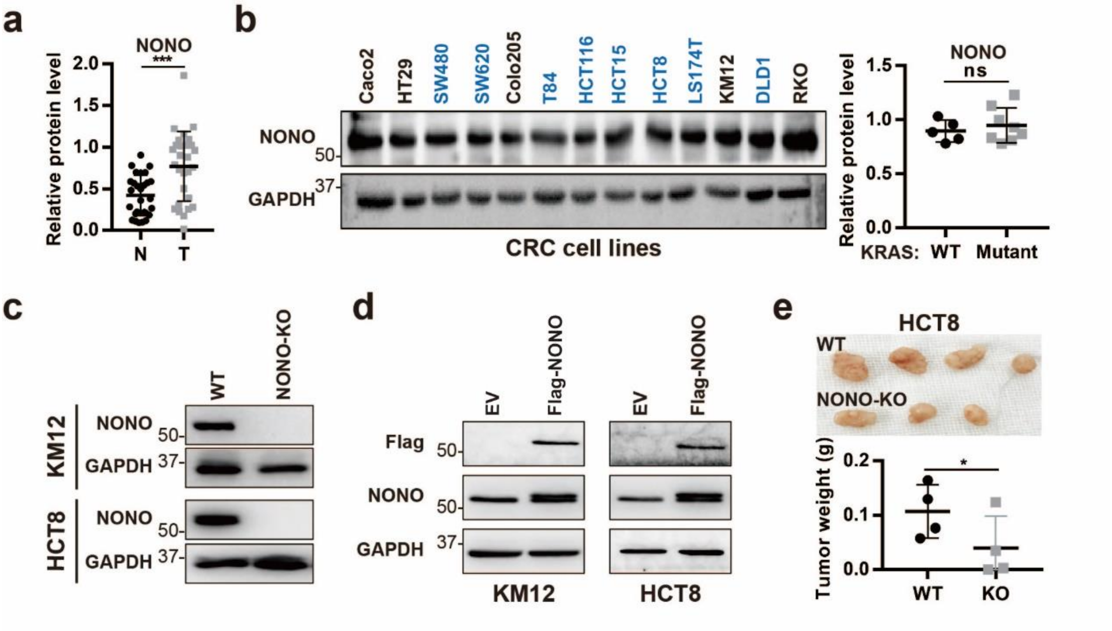

Supplementary Figure S1. NONO is overexpressed in CRC. a The intensity of NONO protein bands were analyzed by Image J. **b** NONO protein expression level is similar between *KRAS* WT and mutated CRC cell lines. NONO protein was detected using western blotting assay (left panel), and the intensity of protein bands were analyzed by Image J. The *KRAS* mutated cell lines were shown as blue color. **c** Construction of NONO KO CRC cells. Western blotting analysis of NONO expression in CRISPR/Cas9-mediated NONO knockout KM12 and HCT8 cells. **d** NONO is overexpressed in CRC cells. EV or Flag-NONO were transfected into KM12 and HCT8 cells for 24 h, and subjected to western blotting analysis. **e** NONO knockout inhibits xenograft growth. HCT8 WT or NONO KO cells (1×10^6) were injected into nude mouse (n = 4) for 21 days before tumor weight examination. EV, empty vector (pCDH-CMV) transfected cells; NONO-OE, NONO (pCDH-CMV-Flag-NONO) transfected cells. ns, no significance. *P<0.05, ***P<0.001; ns, no significance.

Supplementary Figure S2

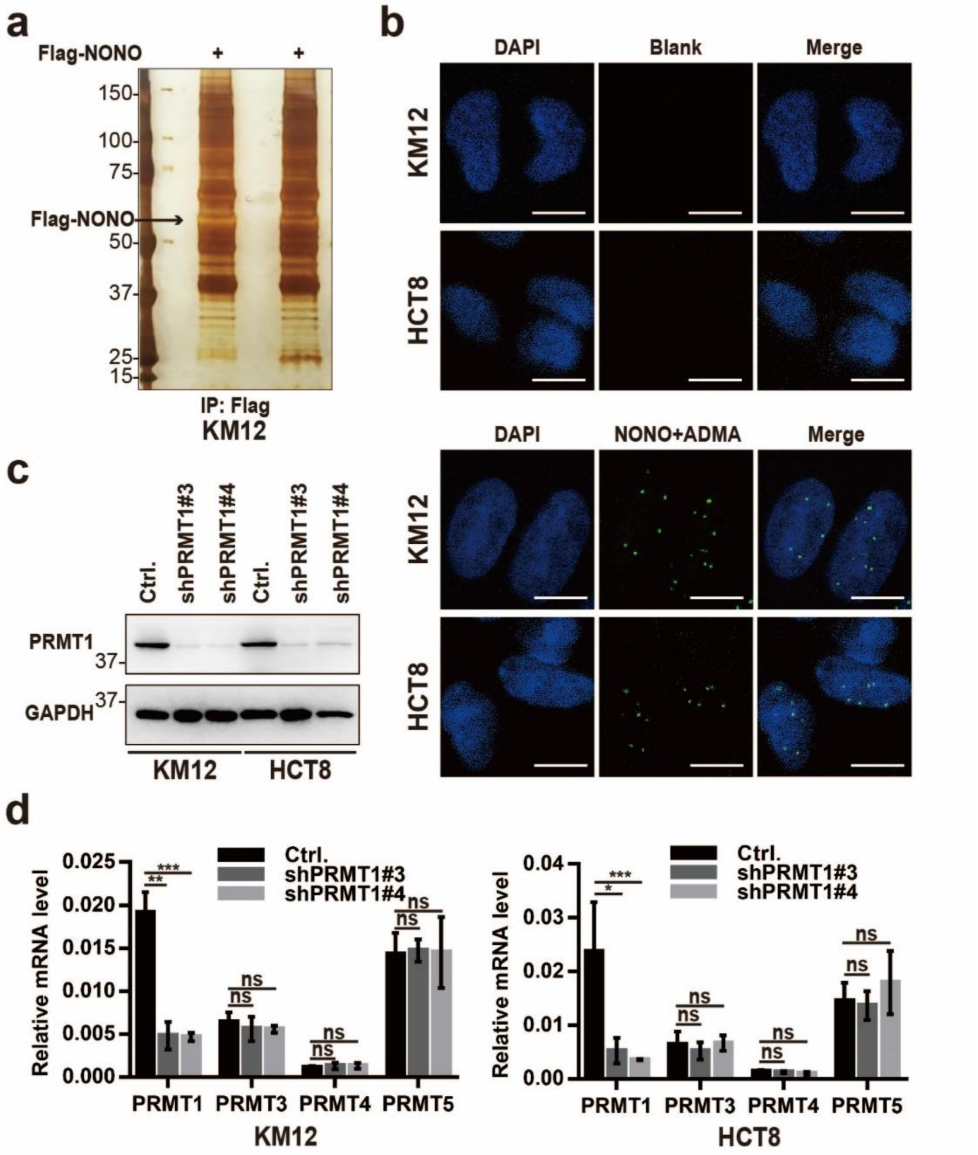

Supplementary Figure S2. PRMT1 mediates NONO arginine methylation in CRC cells. a Silver staining of purified Flag-NONO protein. Flag-NONO was immunoprecipitated from control and PRMT1-silenced KM12 cells, and subjected to western blotting analysis and silver staining. **b** NONO is asymmetric arginine dimethylated in CRC cells. Duolink PLA was performed using anti-NONO and -ADMA antibodies. The positive signal was presented as green fluorescence spots. Blank indicated as a negative control without primary antibodies incubation. Scale bar, 10 μm. **c** Construction of PRMT1 knockdown cells. **d** qPCR assay was conducted to detect the mRNA expression level of PRMTs genes. *P<0.05, **P<0.01, ***P<0.001; ns, no significance.

Supplementary Figure S3

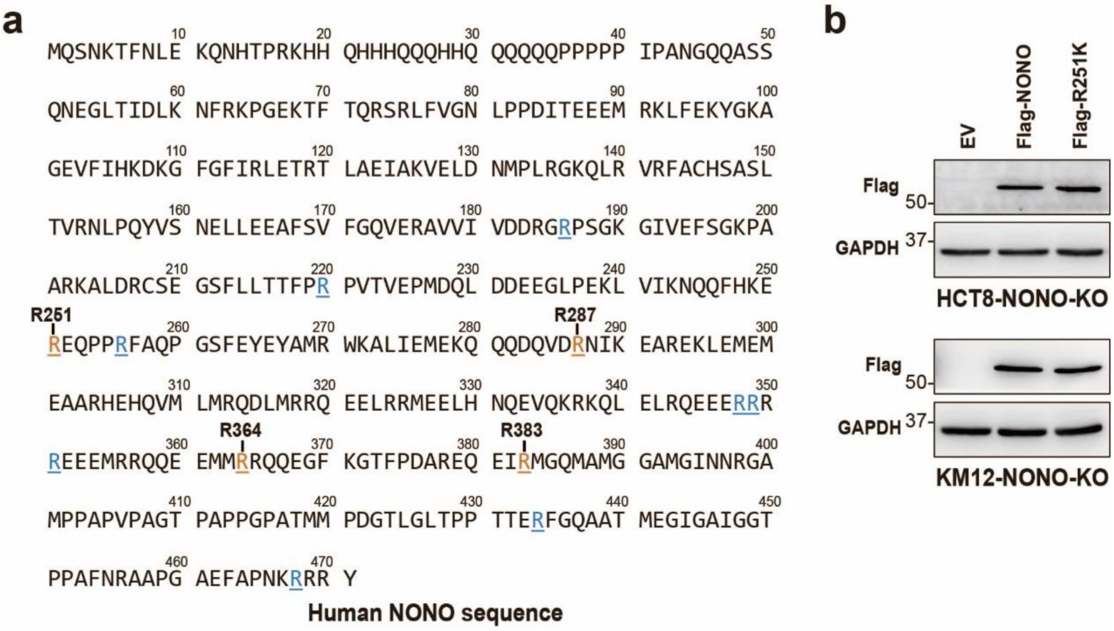

Supplementary Figure S3. PRMT1-mediated methylation at R251 is required for the oncogenic function of NONO. a The amino acid sequence of NONO. All methylated R residues were underlined, and R251, R287, R364, and R383 were presented in control, but not in PRMT1 knockdown cells. **b** Flag-tagged WT or R251K of NONO were re-expressed inKM12 and HCT8 NONO knock out cells.

Supplementary Figure S4

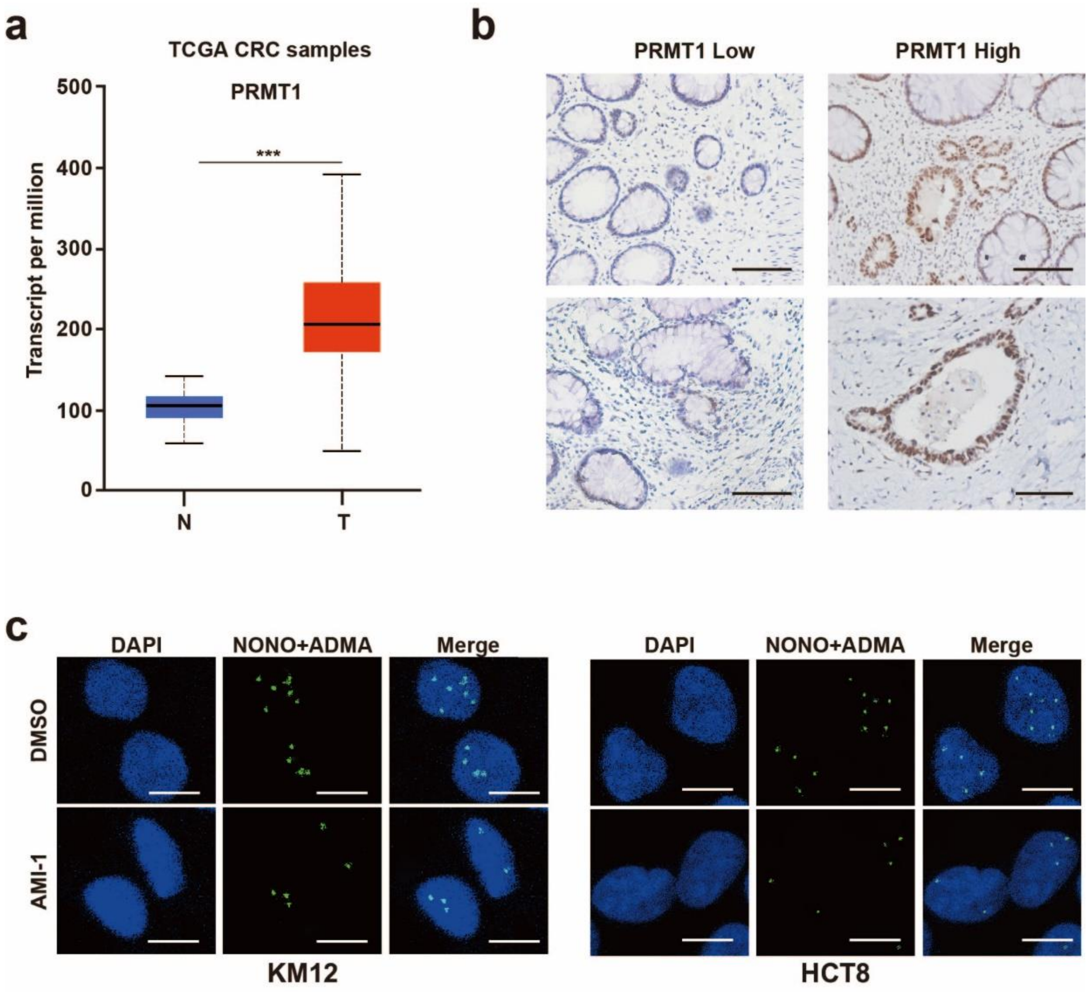

Supplementary Figure S4. PRMT1 is overexpressed in CRC tissues. a PRMT1 mRNA level in TCGA normal (N, n=41) and tumor (T, n=286) samples. **b** The protein expression of PRMT1 was evaluated by IHC staining. The 97 patients were divided into PRMT1 low (n = 61) and high (n = 36) subgroups. Scale bar, 1200 μm. **c** Representative Duolink PLA images of KM12 and HCT8 cells treating with DMSO or AMI-1. Scale bar, 10 μm. ***P<0.001.

Supplementary Figure S5

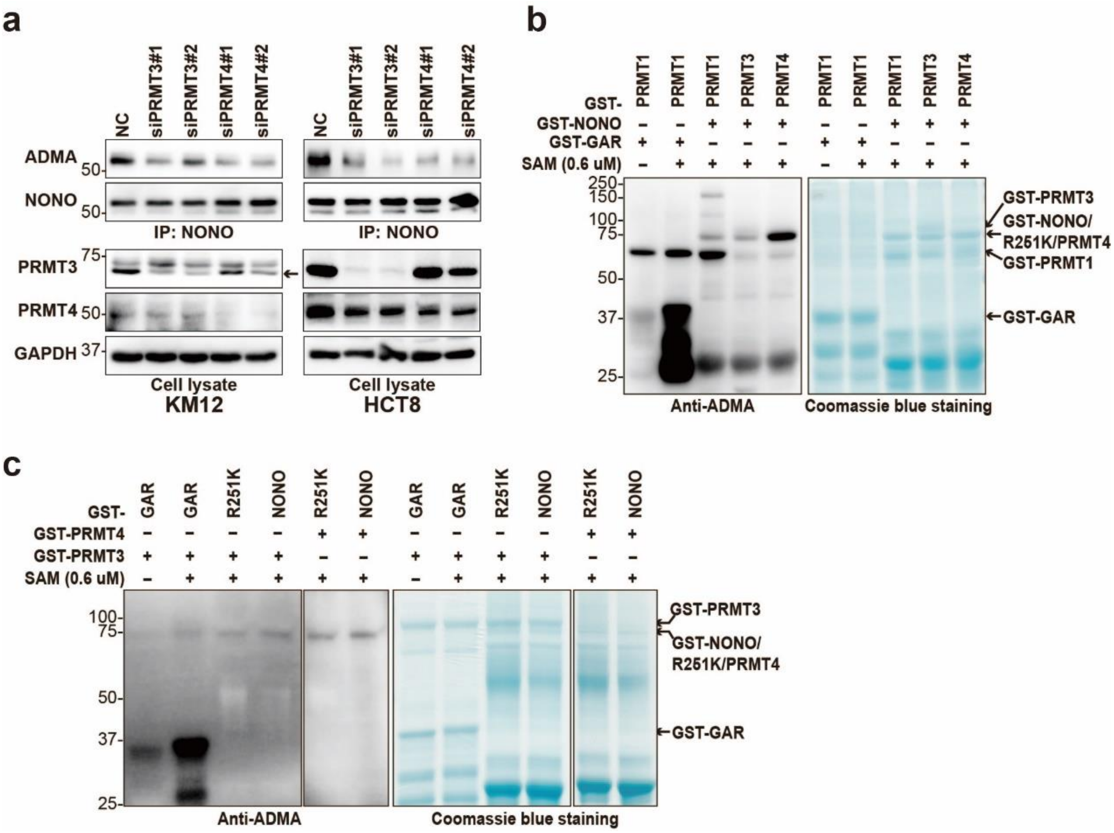

Supplementary Figure S5. PRMT3 and PRMT4 is not responsible for arginine methylation of NONO on R251. a PRMT3 and PRMT4 methylate NONO in vivo. Silencing of PRMT3 and PRMT4 reduced NONO aDMA. Endogenous NONO was immunoprecipitated from control, PRMT3- and PRMT4-silenced KM12 and HCT8 cells, and subjected to western blotting analysis. **b** PRMT3 and PRMT4 methylate NONO in vitro. **c** PRMT3 and PRMT4 do not methylate NONO at R251. For b and c, GST-tagged GAR, NONO, and R251K were incubated with purified GST-PRMT1/PRMT3/PRMT4 in the presence or absence of 0.6 μM SAM respectively. Protein samples were separated on SDS-PAGE and subjected to western blotting analysis and coomassie blue staining.

Supplementary Figure S6

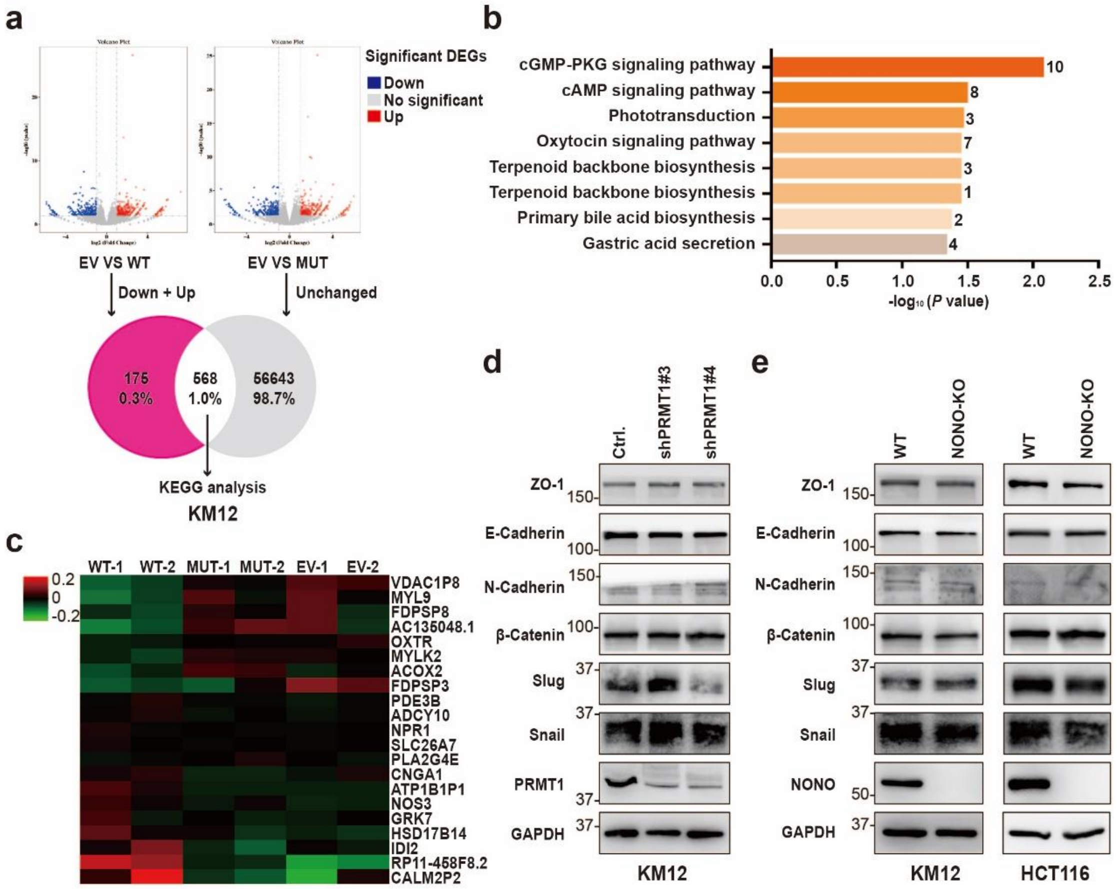

Supplementary Figure S6. The pathways regulate by NONO arginine methylation. a The schematic diagram of genes use for KEGG analysis. **b** The KEGG analysis of significant pathways ($P < 0.05$). **c** The heat map of the specific genes involved in cAMP pathways. For a-c, EV-1/2, KM12-NONO-KO cells transfected with pCDH-GFP; WT-1/2, KM12-NONO-KO cells transfected with pCDH-GFP-NONO; MUT-1/2, KM12-NONO-KO cells transfected with pCDH-GFP-NONO-R251K. **d, e** *PRMT1* silencing or *NONO* knockout do not alter EMT-related markers' expression. EMT-related protein expression was detected by western blotting.

Supplementary Table S1. Correlations between expression of NONO and clinicopathological characteristics of CRC patients

Characteristics		NONO		P
		Low	High	
Age, years	<57	27	18	0.117
	≥57	21	27	
Gender	Male	28	30	0.407
	Female	20	15	
cT	T2	1	3	0.179
	T3	35	25	
	T4	12	17	
cN	N0	8	12	0.501
	N1	13	11	
	N2	27	22	
TNM stage	II	0	3	0.162
	III	8	9	
	IV	40	33	
Differentiation	Low	12	19	0.002*
	Intermediate	22	25	
	High	14	1	
Metastasis[a]	No	38	32	0.368
	Yes	10	13	

*Statistically significant

[a]Metastasis occurred after surgery.

Supplementary Table S2 Arginine methylation sites of NONO in colorectal cancer cells.

Methylation site	Amino acid sequence	Score
**The arginine methylation sites of NONO in KM12 control cells**		
R168	GRPSGGIVEFSGKPAAR	33
R220	CSEGSFLLTTFPRPVTVEPMDQLDDEEGLPEK	3
R251	EREQPPR	13
R256	EQPPRFAQPGSFEYEYAM	32
R287	ALIEMEKQQQDQVDR	0
R348/349	QEEERR	6
R351	REEEMR	2
R364	QQEEMMR	3
R383	EQEIRMGQMAMGGAMGINNR	1
R434	GAMPPAPVPAGTPAPPGPATMMPDGTLGLTPPTTER	4
R468	AAPGAEFAPNKR	17
**The arginine methylation sites of NONO in KM12 PRMT1-silenced cells**		
R168	GRPSGGIVEFSGKPAAR	33
R220	CSEGSFLLTTFPRPVTVEPMDQLDDEEGLPEK	3
R256	EQPPRFAQPGSFEYEYAM	32
R348/349	QEEERR	6
R351	REEEMR	2
R434	GAMPPAPVPAGTPAPPGPATMMPDGTLGLTPPTTER	4
R468	AAPGAEFAPNKR	17

Individual ions score >4 indicate identity or extensive homology ($P<0.05$)

Supplementary Table S3. Correlations between expression of PRMT1 and clinicopathological characteristics of CRC patients

Characteristics		PRMT1		P
		**Low**	**High**	
Age, years	<56	33	12	0.059
	≥56	28	24	
Gender	Male	37	24	0.554
	Female	24	12	
cT	T2	1	3	0.155
	T3	43	20	
	T4	17	13	
cN	N0	13	7	0.757
	N1	16	12	
	N2	32	17	
TNM stage	I	0	3	0.040*
	II	13	4	
	III	48	29	
Differentiation	Low	15	13	0.231
	Intermediate	35	19	
	High	11	4	
Metastasis[a]	No	50	28	0.615
	Yes	11	8	

*Statistically significant

[a]Metastasis occurred after surgery.

Supplementary Table S4. Sequences of DNA oligonucleotides

Name	Sense Strand/Sense Primer (5'-3')	Antisense Strand/Antisense Primer (5'-3')
**Primers for qPCR**		
PRMT1	CTTTGACTCCTACGCACACTT	GTGCCGGTTATGAAACATGGA
PRMT3	CAGAACCTGCTCGTCATCTACT	TTCCACACCCAACATCCAAAA
PRMT4	CAGCAGAACATGATGCAGGAC	CGCGTAGATTTTCCGTGCTC
PRMT5	TCAGGAAGATAACACCAACCTGG	AGCCACTGCAATCCTCTTACTAT
PRMT6	TACCGCCTGGGTATCCTTCG	CCTGTTCCGGCAACTCTACA
PRMT8	TGTTTGGGATCGAATGCTCCA	TGGCAAAGATCACCGTGTTGA
NONO	GGCAGGCGAAGTCTTCATTCA	TGGCAATCTCCGCTAGGGT
U6	CGGCAGCACATATAC	TTCACGAATTTGCGTGTCAT
**Primers for site mutation**		
NONO-R251K	AAGGAGCAGCCACCCAGATTTGCACAGC	TTCCTTGTGAAATTGCTGGTTTTTTATAAC
NONO-R287K	AAGAACATCAAGGAGGCTCGTGAGAAGC	GTCCACTTGGTCCTGCTGCTGCTTCTCCAT
NONO-R364K	AAGCGACAGCAGGAAGGATTCAAGGGAA	CATCATTTCTTCTTGCTGCCGCCGCATCTC
NONO-R383K	AAGATGGGTCAGATGGCTATGGGAGGTG	AATCTCCTGCTCTCTCGCATCAGGGAAGGT

Supplementary Table S4. Sequences of DNA oligonucleotides (Continued)

Name	Sense Strand/Sense Primer (5'-3')	Antisense Strand/Antisense Primer (5'-3')
**Primers for cloning (restriction enzyme sites are underlined)**		
Flag-NONO	TGA<u>GAATTC</u>GCCACCATGGATTACAAGGATGACGACG ATAAG CAGAGTAATAAAACTTT	TCA<u>GCGGCCGC</u>TTAGTATCGGCGACGTTTGT
Myc-NONO	TGA<u>GAATTC</u>GCCACCATGGAGCAGAAACTCATCTCTGA AGAGGATCTG CAGAGTAATAAAACTTT	TCA<u>GCGGCCGC</u>TTAGTATCGGCGACGTTTGT
Flag-PRMT1	TGA<u>GAATTC</u>GCCACCATGGATTACAAGGATGACGACG ATAAG GCGGCAGCCGAGGCCGC	TCA<u>GCGGCCGC</u>TCAGCGCATCCGGTAGTCGG
HA-PRMT1	TGA<u>GAATTC</u>GCCACCATGTACCCATACGATGTTCCAGA TTACGCTGCGGCAGCCGAGGCCGC	TCA<u>GCGGCCGC</u>TCAGCGCATCCGGTAGTCGG
Flag-PRMT1-PRD+CD	TGA<u>GAATTC</u>GCCACCATGGATTACAAGGATGACGACG ATAAG GCGGCAGCCGAGGCCGC	TCA<u>GCGGCCGC</u>TCACTCGCACAGCTGGCCCT
Flag-PRMT1-CD	TGA<u>GAATTC</u>GCCACCATGGATTACAAGGATGACGACG ATAAG AAAGATTACTACTTTGA	TCA<u>GCGGCCGC</u>TCACTCGCACAGCTGGCCCT

Supplementary Table S4. Sequences of DNA oligonucleotides (Continued)

Name	Sense Strand/Sense Primer (5’-3’)	Antisense Strand/Antisense Primer (5’-3’)
**Primers for cloning (restriction enzyme sites are underlined)**		
Flag-PRMT1-CD+POD	TGAGAATTCGCCACCATGGATTACAAGGATGACGACGATAAG AAAGATTACTACTTTGA	TCAGCGGCCGCTCACTCGCACAGCTGGCCCT
(GST)-NONO	TGAGGATCCGCCACCCAGAGTAATAAAACTTTTAA	TCAGAATTCGTATCGGCGACGTTTGTTTG
(GST)-PRMT1	TGAGGATCCGCCACCGCGGCAGCCGAGGCCGCGAA	TCAGAATTCTCAGCGCATCCGGTAGTCGG
(GST)-GAR	TGAGGATCCGCCACC AAGCCAGGATTCAGTCCCCG	TCAGAATTCTCAAGCTTGGAGCGGAAGGG
**Primers of shRNA or sgRNA**		
shPRMT1#3	CCGGGCAACTCCATGTTTCATAACTCGAGTTATGAAACATGGAGTTGCTTTTTG	AATTCAAAAAGCAACTCCATGTTTCATAACTCGAGTTATGAAACATGGAGTTGC
shPRMT1#4	CCGGCCGGCAGTACAAAGACTACAACTCGAGTTGTAGTCTTTGTACTGCCGGTTTTTG	AATTCAAAAACCGGCAGTACAAAGACTACAACTCGAGTTGTAGTCTTTGTACTGCCGG
NONO-sgRNA	ATCGTCTCTCACCGAGGGGAGAACTCCGATTGATGTTTTAGAGCTAGGCCAACATG	ACTGGATCCAAAAAAAGCACCGACTCGGTGCCAC
